# Investigation of the Fire Performance of Polyamide 6-Based Composites with Halogen-free Flame Retardants and Synergistic Materials

**DOI:** 10.1021/acsomega.2c02018

**Published:** 2022-08-09

**Authors:** Tugce Uysalman, Merve Sağlam, Kerim Eraslan, Hava Cekin, Yoldas Seki, Lutfiye Altay, Mehmet Sarikanat

**Affiliations:** † İzmir Eğitim Sağlık Sanayi Yatırım A.Ş., Turgutlu 45400, Manisa, Turkey; ‡ Faculty of Science, Dokuz Eylul University, Buca 35220, Izmir, Turkey; § Mechanical Engineering Department, Ege University, Bornova 35040, Izmir, Turkey

## Abstract

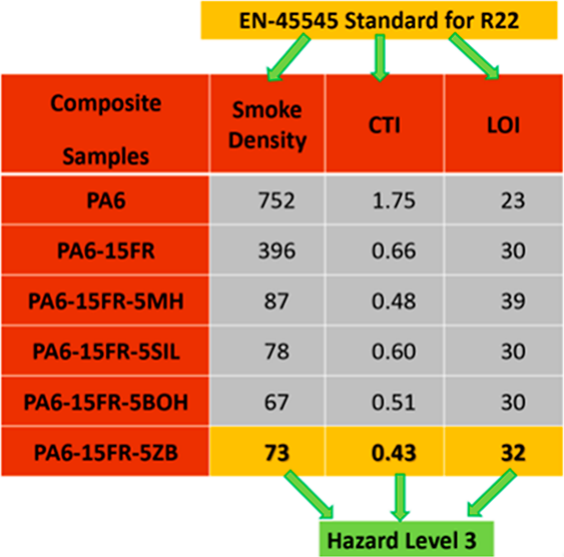

In this study, halogen-free flame retardants and metal synergist materials were used to enhance the flammability of PA6. PA6-based composites including various fractions of additives were manufactured using a twin-screw extruder and an injection molding machine. Mechanical, thermal, physical, morphological, and flame retardant properties were investigated with several characterization methods. The study aims to meet R22 requirements based on the EN45545 standard for fire protection of railway vehicles, according to which limiting oxygen index (LOI), smoke density, and conventional index of toxicity (CIT) values under HL3 hazard levels have to be min 32%, max 300, and max 1.5, respectively. 15FR-2MH, 15FR-5MH, 15FR-1MH-1ZB, 15FR-1MH-1BOH, and 15FR-1MH-1SIL composites exhibited both the required smoke density, CIT, and LOI values for R22. It can be said that hybrid synergists provide all requirements according to the R22-EN45545 standard. Instead of using 15FR-2MH, 15FR-1MH-1BOH led to a lower smoke density value for PA6.

## 1 Introduction

Polymeric composites have been introduced to satisfy the need to achieve faster, safer, and eco-friendly solutions for various industries where being lightweight and having flammability, low cost, recyclability, functionality, and so forth are the requirements. These industries, including aerospace, defense, automotive, railway, electric electronics, and so forth, are seeking materials that are alternatives to metals with functional properties. The selection of materials that are used in different parts of the railway vehicles plays an important role in achieving better transportation for the railway industry and governing bodies. The usage of polymeric composites supports longer material life due to lower degradation, lightweight vehicles, and cost-efficient applications. The selection of the appropriate polymer type depends on various aspects that must address engineering standards. Polyamide 6 has been widely used because of its high thermal stability, high mechanical standards, excellent strength and hardness features, and chemical resistance. However, polyamide 6 (PA6) is flammable and has a low limiting oxygen-index value (LOI), which restricts its applications in especially electrical and other industries.^1−3^ Therefore, the flammability properties of PA6 such as LOI have to be improved for use in railway applications. In order to standardize the flammability results in an engineering manner, materials used in railway applications must satisfy predetermined hazard levels (HL1, HL2, and HL3). These hazard levels have been introduced with the EN45545-2 standard specifically for railway applications. The data obtained from the limited oxygen index, flue gas density, and smoke toxicity tests are classified under each hazard level with the corresponding values.^4−7^ According to the EN45545-2 standard (fire protection railway vehicles), for the requirement set of R22, which shows a supply line system and high-power devices (isolators, current and voltage transformers, contractors, etc.), the requirements for LOI, smoke density, and conventional index of toxicity (CIT) values under HL3 hazard levels have to be min 32%, max 150, and max 0.75, respectively. In order to achieve these requirements, the combination of materials having different flame retardant mechanisms is necessary. It is known that in order to achieve higher LOI values, synergistic materials such as metal salts and minerals could be incorporated into PA6 composites. Some of them are zinc borate(ZB), Mg(OH)_2_(MH), and boehmite (BOH). ZB is an effective inorganic flame retardant and is used in smoke suppression and to promote charring.^8^ ZB decomposes and generates a covering layer on the polymer. This layer prohibits emitted gases from escaping and prevents oxidation.^9−11^ MH, which is known for its smoke suppression effect, could dilute flammable gas and form a layer of adiabatic material on the interface of the plastic material and flame.^12,13^ Moreover, BOH increased the LOI values of polymers and played a role in the smoke suppression effect.^14,15^ High molecular-weight siloxane (SIL), which is known for its flame retardant synergist and smoke suppressant properties, can be used as a flame retardant material.

In this study, in order to achieve R22 requirements according to the EN45545 standard, polyamide was compounded by using aluminum diethyl-phosphinate (DEPAL) and synergists such as ZB, MH, SIL, and BOH. To our knowledge, the effect of hybrid synergists, which are used in this study, together with DEPAL in PA6 formulations on flame retardant properties has not been studied up to now. PA6 was compounded with different amounts of additives and injections molded into test specimens. LOI, glow wire flammability index, UL94, cone calorimetry, thermal, morphological, physical, and mechanical analyses of the PA6-based composites were performed.

## 2 Materials and Methods

### 2.1 Materials

Trade names and specific density values of synergistic agents used in this study are given in Table 1. PA6 with the trade name polyamide 2.7 was supplied from DOMO Chemicals. A flame retardant additive, DEPAL, was supplied from Clariant under the trade name Exolit OP 1230. The synergistic agents, which are magnesium hydroxide, SIL (AP 1142A), boehmite, and zinc borate, were supplied by brucite, Auser Polimeri, tor chemicals, and melos kimya, respectively.

**Table 1:** Trade Names and Density Values of Synergistic Agents

synergistic agent	trade name	specific density (g/cm^3^)
magnesium hydroxide	Ecopiren 3,5 C	2.4
SIL	Silmaprocess AA1142A	2.4
boehmite	TOR Brite	3.0
zinc borate	ZB 467	2.7

### 2.2 Production of Composite Materials

Production of composite materials is performed by using a twin-screw extruder device (Leistritz Extruder Corporation Model ZSE 27 MAXX). Sample codes of the PA6-based composites with various weight fractions of flame retardants and synergistic agents are given in Table 2. After producing the composites, the samples are prepared by injection molding (Bole model BL90EK) for sampling according to the required standards.

**Table 2:** Sample Codes of the Samples

sample code	flame retardant, FR	magnesium hydroxide, MH	zinc borate, ZB	boehmite, BOH	siloxane, SIL
15FR	15	0	0	0	0
15FR-1MH	15	1	0	0	0
15FR-2MH	15	2	0	0	0
15FR-5MH	15	5	0	0	0
15FR-1SIL	15	0	0	0	1
15FR-2SIL	15	0	0	0	2
15FR-5SIL	15	0	0	0	5
15FR-1BOH	15	0	0	1	0
15FR-2BOH	15	0	0	2	0
15FR-5BOH	15	0	0	5	0
15FR-1ZB	15	0	1	0	0
15FR-2ZB	15	0	2	0	0
15FR-5ZB	15	0	5	0	0
15 FR-1MH-1ZB	15	1	1	0	0
15FR-1MH-1BOH	15	1	0	1	0
15FR-1MH-1SIL	15	1	0	0	1

### 2.3 Characterization

#### 2.3.1 Density

Density measurement of the specimens was conducted according to ASTM D792 using the densimeter MD-200S. The density of each composite was obtained by calculating the average of the density values of three tests.

#### 2.3.2 Differential Scanning Calorimetry Analysis

Differential scanning calorimetry (DSC) Q20 (TA Instruments Inc., DSCQ20) was used for DSC analysis. Measurements were carried out within the temperature range of 50 and 900 °C at a rate of 10 °C/min. Each sample (5–6 mg) was weighed out in a standard aluminum pan. The sealed pan was scanned at a heating rate of 10 °C/min under a nitrogen atmosphere. The degree of crystallinity was determined by using the following eq 1
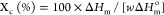
1where Δ*H*_m_ is the melting enthalpy of PA6 in the composites, *w* is the weight fraction of polymer, and Δ*H*_m_^o^ is the melting enthalpy of fully crystalline PA6 (188 J/g).^16^

#### 2.3.3 Thermogravimetric Analysis

TG analyses were made on TA Instrument Q600 model simultaneous thermal analysis equipment. Measurements were recorded at a heating rate of 10 °C/min from room temperature to 600 °C in a nitrogen atmosphere.

#### 2.3.4 Thermo-Mechanical Analysis

The thermal expansion coefficients (CTE) of PA6-based composites were determined using a thermo-mechanical analyzer (TA Instruments, Inc., TMA 400) using the expansion mode. Specimens (10 mm × 8 mm × 4 mm) were heated from −30 to 100 °C at a rate of 5 C min^–1^. In order to provide good contact between the probe and the specimen, a pre-load force of 0.02 N was applied through the thickness direction.

#### 2.3.5 Tensile Test

The tensile properties of the composites were investigated using the Hegewald–Peschke Inspect 20 universal testing machine at a cross-head speed of 50 mm/min according to the ISO 527 standard.

#### 2.3.6 Flexural Test

Three-point bending tests were conducted to characterize the flexural properties of the PA6 composite plates by following the ISO 178 standard. The tests were carried out at a constant cross-head speed of 2 mm/min and a span length of 64 mm. Average values of five tests were recorded.

#### 2.3.7 Izod Impact Test

Notched and un-notched Izod impact testing was conducted using ISO 180 on the Izod/Charpy impact tester. The specimens of 4 mm thickness were cut into 10 mm in width and 80 mm in length. A 2.55 mm notch was made using a CNC machine. Tests were operated at room temperature with a 5J Stricker and a span length of 70 mm.

#### 2.3.8 Flame Retardant Properties

##### 2.3.8.1 UL94 Vertical Burning Test

UL94 flame rating of the composites was tested in the ATLAS horizontal and vertical burning tester according to the UL94 standard. The specimens used were 125 × 13 × 1.5 mm^3^ and 125 × 13 × 3 mm^3^.

##### 2.3.8.2 Limiting Oxygen Index

The LOI values were obtained using an LOI instrument (Fire Testing Technology (FTT, UK)) according to ISO 4589, and the specimen dimensions were 80 mm × 10 mm × 4 mm.

##### 2.3.8.3 Glow Wire Flammability Index

Glow wire tests of the composites were conducted in a test cabinet (Fire Testing Technology, UK) using samples of 60 × 90 × 2.5 mm^3^ according to the IEC 60695 standard. The specimens are put into contact with the glowing wire heated at a determined temperature ranging from 550 to 960 °C. The glow wire flammability index (GWFI) was determined.

##### 2.3.8.4 Cone Calorimetry Test

The combustion test was performed on the cone calorimeter (Fire Testing Technology, UK) in a horizontal mode according to ISO 5660 using an external heat flux of 50 kW m^–2^ with 100 mm × 100 mm × 3 mm specimens. The flammability parameters include the time to ignition (TTI), peak heat release rate (PHRR), total smoke rate (TSR), total heat release (THR), time of peak heat release rate (tPHRR), fire growth rate (FIGRA), effective heat combustion (EHC), and maximum average rate of heat emission (MAHRE). Another important parameter during combustion produced by fire is the amount of CO and CO_2_ released from the burned products due to the fact that a great release could cause anoxia conditions.^17^ It is known that death in a fire disaster is mainly owing to the production of CO.^18^ Therefore, in relation to the toxicity, the concentrations of toxic gases (CO_2_ and CO) and CIT were also quantified at 480 s.^17^

#### 2.3.9 Scanning Electron Microscopy Observation

The morphological characterization of PA6-based composites was conducted by scanning electron microscopy (SEM, Carl Zeiss 300VP, Germany) operated at 7.5 kV. Gold was deposited on the surface of the fracture surfaces of PA6 and its composite specimens by using a plasma sputtering apparatus.

## 3 Results and Discussion

### 3.1 Density

**Figure 1:**
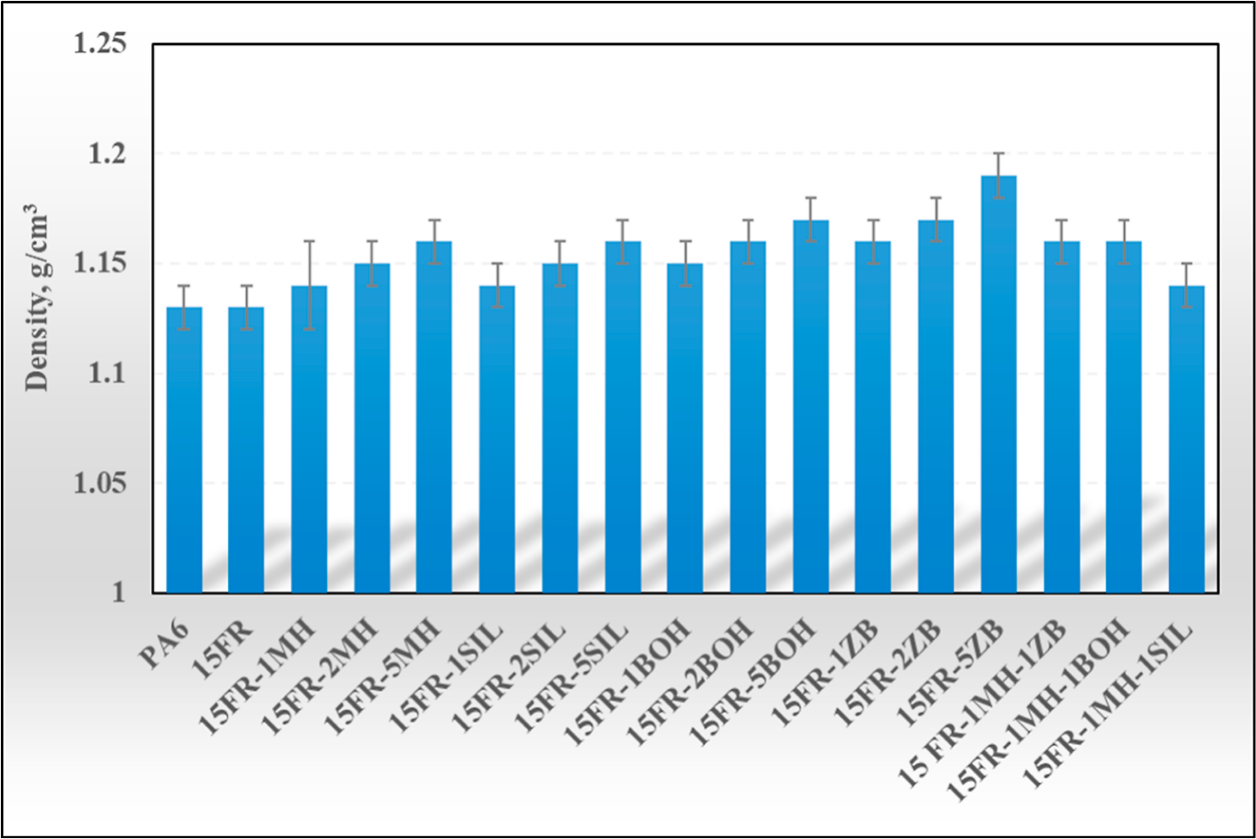
Density values of the composites.

In addition to their flame retardant performance, in applications where weight is critical, the density of the composite material becomes more important, and the lightest ones among the synergistic agents can be preferred more. For that reason, the density of composites is measured. Results of density measurement of the composites are given in Figure 1 with standard deviations. As it is expected, the 15FR composite has the lowest density value, which is 1.13 g/cm^3^. As it is seen, density values are increased by increasing the synergistic agent ratio in the composites. In addition to their flame retardant performance, achieving similar performance with low density will provide an advantage in the field of applications where part weight matters.

### 3.2 DSC Analysis

The crystallization temperature (*T*_c_), melting temperature (*T*_m_), melting enthalpy (Δ*H*_m_), crystallization enthalpy (Δ*H*_c_), and crystallinity (*X*_c_) are summarized in Table 3. It was observed from the DSC measurements that the presence of fillers, MH, SIL, ZB, or BOH, does not affect the *T*_m_ of the PA6 matrix, which is about 222 °C. From Table 3, it is seen that the *T*_c_ value of the PA6 increased when 15 wt % FR was used in PA6. When other fillers were added into PA6 with 15FR, it was observed that MH addition caused a decrease in *T*_c_ values of composites. It is expected that introducing a higher number of particles inhibits chain mobility and, thus, retards crystal growth. On the contrary, the *T*_c_ value for 15FR-5BOH is about 186 °C, with an increase of about 2 °C as compared to that of PA6 with 15FR. This result indicates that BOH can act as a nucleation agent in the PA6 matrix, resulting in increased polymer chain mobility and leading to faster crystallization. Fillers, to some extent, may act as nucleating agents, causing a higher crystallization rate than that of neat PA6.^19^ Moreover, it has been reported that faster crystallization arising from increased nucleation could also be due to the presence of impurities incorporated in the matrix during processing.^20^ The addition of SIL to PA6 causes a slight decrease in the crystallinity of PA6. One can say that SIL particles could restrict the growth of PA6, leading to a reduction in crystallinity. Similar behavior has been observed in PA6-based composites with different fillers.^21^

**Table 3:** DSC Data of the Samples

sample	*T*_m_ (°C)	*T*_c_ (°C)	Δ*H*_m_ (J/g)	Δ*H*_c_ (J/g)	*X*_c_ (%)
PA6	222	172	54.2	61.9	28
15FR	220	184	39.3	46.3	24
15FR-1MH	220	182	38.1	49.0	24
15FR-2MH	220	182	41.1	45.3	26
15FR-5MH	220	181	49.0	52.2	32
15FR-1SIL	220	187	40.1	45.3	25
15FR-2SIL	221	186	39.9	47.1	25
15FR-5SIL	220	186	32.4	38.5	21
15FR-1BOH	220	186	44.2	53.4	28
15FR-2BOH	220	186	39.6	45.2	25
15FR-5BOH	220	186	37.2	46.1	24
15FR-1ZB	219	184	37.1	47.5	23
15FR-2ZB	219	185	53.2	65.0	34
15FR-5ZB	220	185	37.0	44.2	24
15 FR-1MH-1ZB	221	183	41.1	52.1	26
15FR-1MH-1BOH	220	185	37.3	48.0	24
15FR-1MH-1SIL	220	182	39.5	50.1	25

### 3.3 TG Analysis

**Figure 2:**
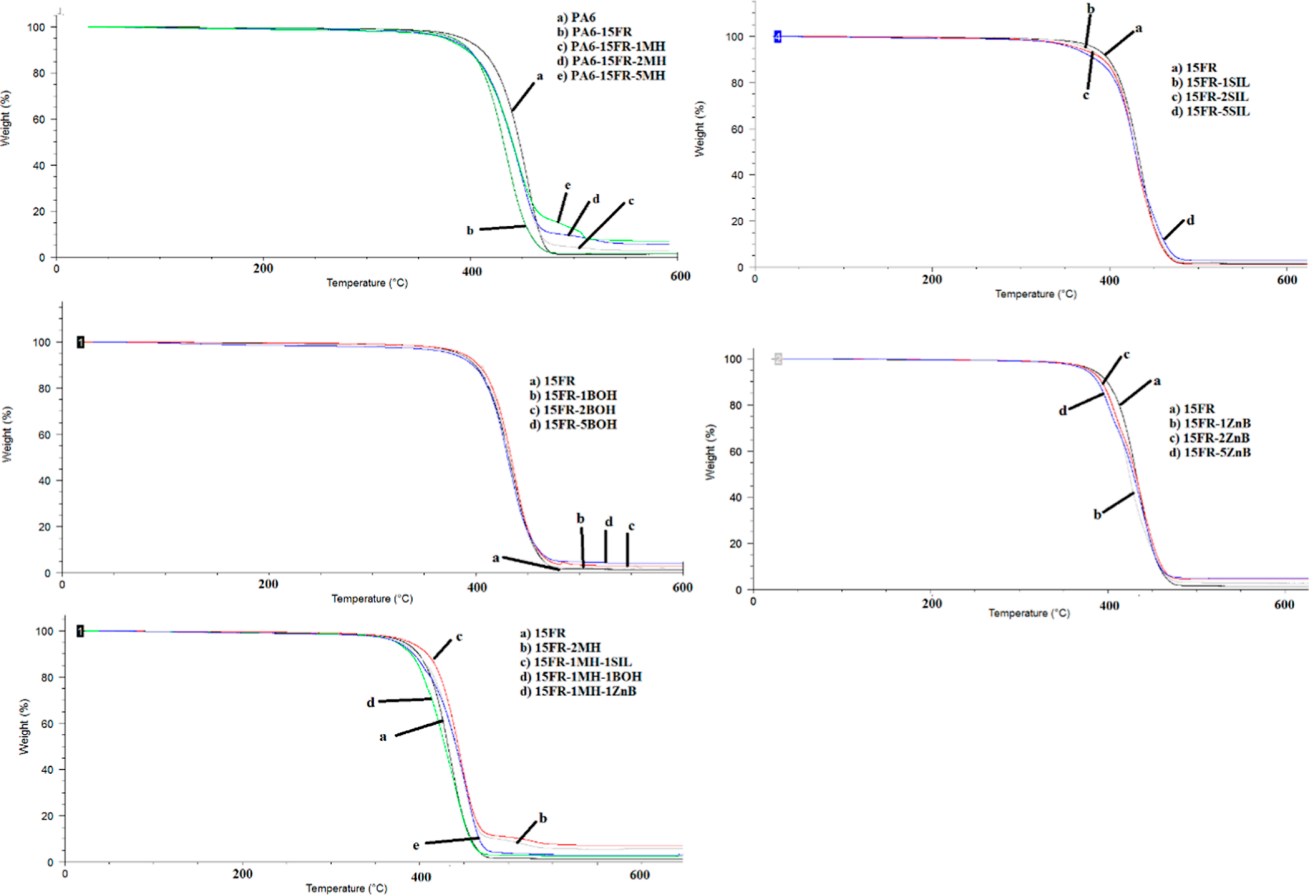
TG curves of PA6-based composites.

TG curves of PA6 and its composites are presented in Figure 2. TG data obtained from Figure 2 are presented in Table 4.

**Table 4:** TGA Data of the Samples

sample	temp at 5% mass loss, °C	*T*_max_, °C	residue (%)
PA6	382	432	1.5
15FR	377	451	3.0
15FR-1MH	377	450	5.6
15FR-2MH	376	445	7.0
15FR-5MH	373	428	1.9
15FR-1SIL	364	429	1.4
15FR-2SIL	359	427	3.2
15FR-5SIL	383	430	2.0
15FR-1BOH	387	438	3.3
15FR-2BOH	377	431	4.4
15FR-5BOH	379	422	3.0
15FR-1ZB	380	438	4.2
15FR-2ZB	376	438	5.0
15FR-5ZB	388	445	7.0
15 FR-1MH-1ZB	374	439	2.4
15FR-1MH-1BOH	375	454	3.5
15FR-1MH-1SIL	382	432	1.5

FR and MH loading into PA6 decreased the temperature at 5% mass loss and maximum degradation temperature. When MH addition increased, the residue values of PA6-15FR increased too. The thermal decomposition of MH takes place, and MgO is formed as a residue.^22^ However, SIL addition into PA5-15FR decreased the temperature at 5% mass loss and maximum degradation temperatures. SIL addition into PA6-15FR has not led to the formation of less residue than MH addition into PA6-15FR. The highest temperatures at 5% mass loss and maximum degradation temperature were obtained by loading 1MH-1SIL and 1MH-1ZB, respectively. It can be noted that the combination of MH with SIL has led to better thermal stability in terms of temperature at 5% mass loss than 2MH and 2SIL. BOH addition into PA6-15FR increased the residue value of PA6-15FR because of the alumina formed by the decomposition of BOH.^23,24^ ZB addition into PA6-15FR has led to the formation of more residue than that of BOH. Zinc borate degrades at 290 °C and dehydrates endothermically, absorbing heat from the vaporized water and locally diluting oxygen and gaseous flammable component processes.^25^

### 3.4 Thermo-Mechanical Analysis

CTE values of PA6-based composites are presented in Table 5. The CTE value of PA6-15FR was determined to be 87.2 μm/m °C. BOH addition into PA6-15FR has led to higher CTE values. As the weight fraction of BOH increases, CTE values also increase. PA6-15FR-1MH-1ZB exhibited the lowest CTE value among the studied composites. It can be said that while the MH addition together with SIL and BOH decreases the CTE value, MH addition together with ZB addition decreases the CTE value. It is known that the decrease in CTE of insulating polymer resin has been observed by adding fillers with a low thermal expansion. This has been attributed to the fact that the mobility of the polymer is restricted by filler materials.^26^ The formation of increased constrained polymers suppresses the thermal expansion of the composites.^27^

**Table 5:** CTE Values of the Samples

sample	CTE (μm/m°C)
PA6	95
PA6-15FR	87.2
PA6-15FR-1MH	72.5
PA6-15FR-2MH	89.1
PA6-15FR-5MH	90.8
PA6-15FR-1SIL	85.0
PA6-15FR-2SIL	86.0
PA6-15FR-5SIL	91.5
PA6-15FR-1BOH	90.7
PA6-15FR-2BOH	94.4
PA6-15FR-5BOH	130.6
PA6-15FR-1ZB	72.1
PA6-15FR-2ZB	96.7
PA6-15FR-5ZB	84.3
PA6-15FR-1MH-SIL	91.7
PA6-15FR-1MH-1BOH	82.8
PA6-15FR-1MH-1ZB	64.8

### 3.5 Mechanical Properties

**Figure 3:**
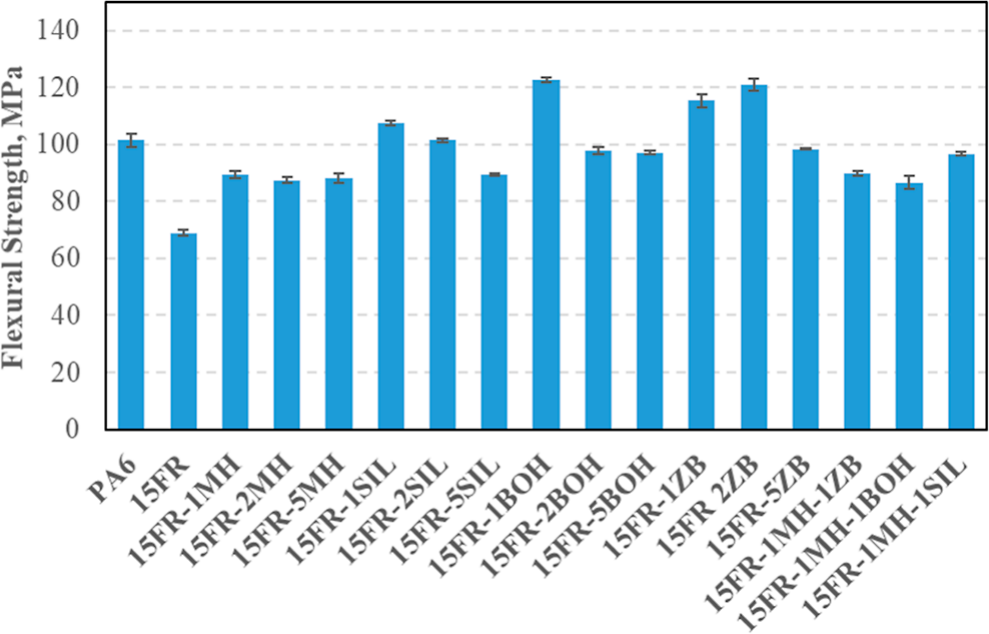
Flexural strength values of PA6 composites.

**Figure 4:**
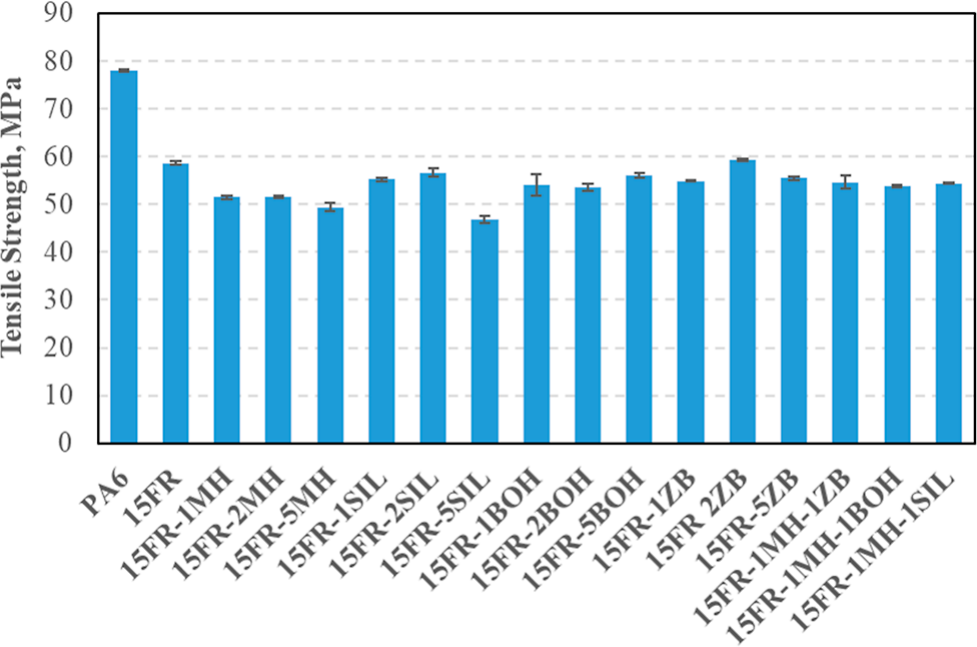
Tensile strength values of PA6 composites.

MH, SIL, BOH, and ZB were added to the formulations containing 15 wt % organic phosphorus-based FR to examine their synergistic effects, and the mechanical test results are shown in Figures 3 and 4, respectively. Flexural and tensile strength values of PA6 (102 and 78 MPa, respectively) were decreased when 15 wt % FR was added into PA6. This can be attributed to the poor compatibility between the flame retardant and the polymer.^28^ When the MH ratio was increased, there was a small change in flexural strength values. Similar behavior was observed in the tensile strength values of MH-added PA6-based composites. However, flexural strength was decreased considerably, when the SIL or BOH ratio was increased from 1 to 5 wt %. The highest flexural strength value was obtained with 1 wt % of BOH. When the hybrid addition of MH-ZB, MH-BOH, and MH-SIL was investigated, it was observed that the highest flexural strength was obtained with the MH-SIL addition. When BOH was added to formulations, tensile strength decreased compared to the 15FR. However, there has been little change in tensile strength for BOH at varying rates of 1, 2, and 5 wt %. It is seen from Figure 4 that the hybrid addition of MH-ZB, MH-BOH, or MH-SIL has not led to a detrimental decrease in tensile strength values compared to those of PA6 composites with 2 wt % ZB, BOH, or SIL. The reason for relatively good mechanical properties could be explained by a uniform distribution of the mineral fillers in the polymer matrix. Madugu et al. (2009) have shown in their previous study that one of the most important points in maintaining mechanical properties is uniform particle distribution.^29^ The reason for the slight decrease in mechanical strength values observed in this study may be due to poor dispersion or agglomeration of fillers in PA6. In the literature, there are studies showing that the interaction between the filler material and the polymer matrix decreases with the increase of the mineral filler ratio in composites, and therefore the mechanical strengths decrease.^30,31^

**Figure 5:**
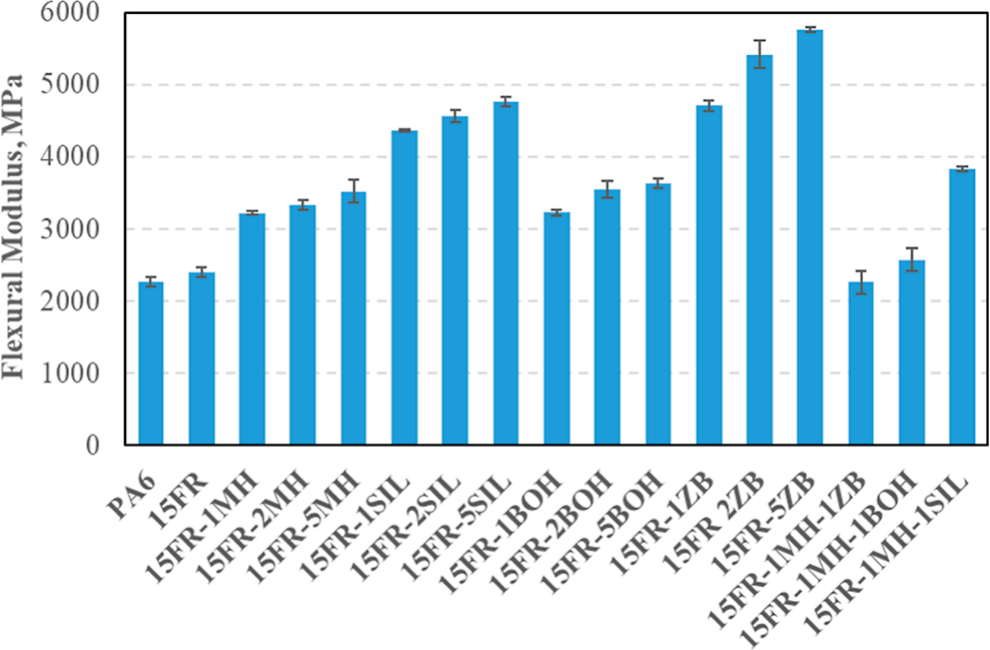
Flexural modulus of PA6 composites.

**Figure 6:**
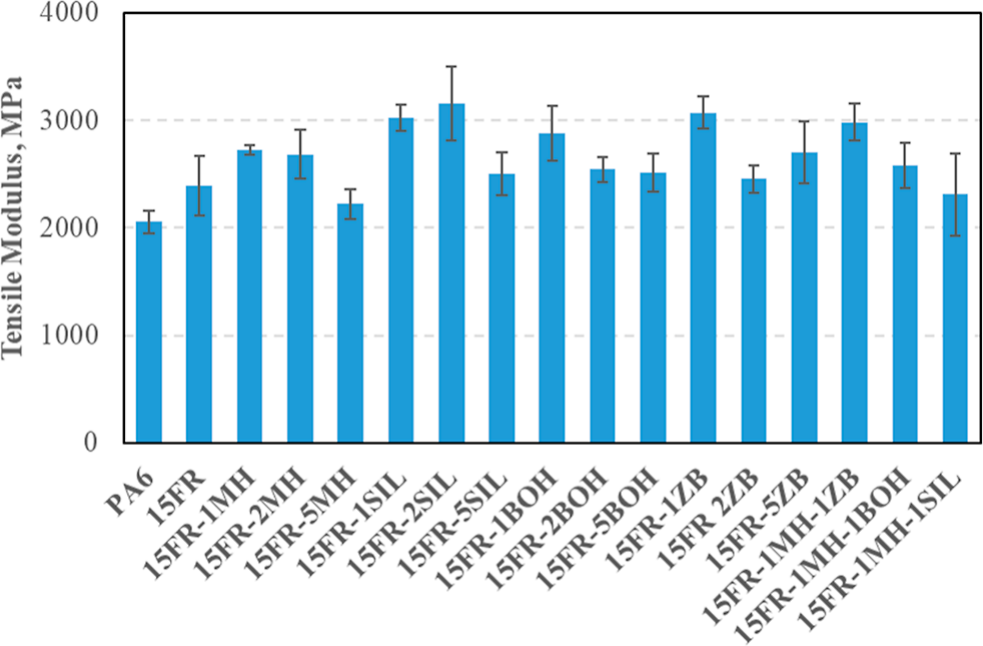
Tensile modulus of flame retardant PA6 composites.

Flexural and tensile modulus values of PA6-based composites are given in Figures 5 and 6, respectively. Among fillers, MH addition has not changed the modulus values; however, when 1–2–5 wt % of SIL was added into the composites, flexural modulus was decreased with the increasing weight fraction of SIL. Similar flexural modulus values were obtained with BOH addition. Adding 5 wt % ZB into the composite increased the flexural modulus up to 5762 MPa, which showed the highest value of flexural modulus of PA6 composites. When it was focused on that, the hybrids MH-ZB and MH-BOH showed similar flexural modulus values being lower than the value of the MH-SIL hybrid.

It was observed from Figure 6 that synergist (MH, SIL, ZB, or BOH) addition caused a slight increase in tensile modulus compared to 15FR at all weight fractions. This could be explained by the stiffness of synergist particles that have a higher value than the polymeric matrix.^31^

**Figure 7:**
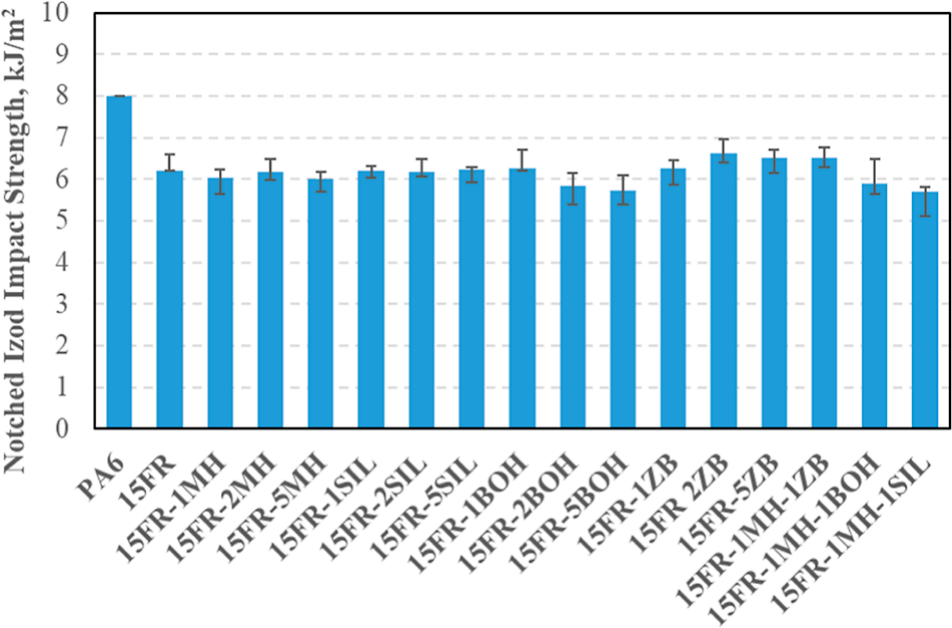
Izod-notched impact strength of PA6 and its composites.

Izod-notched impact strength values of composites are given in Figure 7. Flame retardant addition decreased the Izod-notched impact strength to 6 kJ/m^2^ from 8 kJ/m^2^. Using synergists with 15FR resulted in increased Izod-notched impact strength values. The highest value was obtained when 2% of ZB was used in composites. The increase in the notched impact values with the added filling material can be explained by the fact that as the composite becomes more rigid, it improves the ability to absorb and distribute the energy applied to it and has high impact energy to break the composite.^32^ Guo et al. (2005), in their study on polypropylene/carbonate composites, similarly found that the impact strength increased with the increasing filling ratio, but later, the impact value decreased, although the filling ratio increased.^33^

### 3.6 HDT Vicat

**Figure 8:**
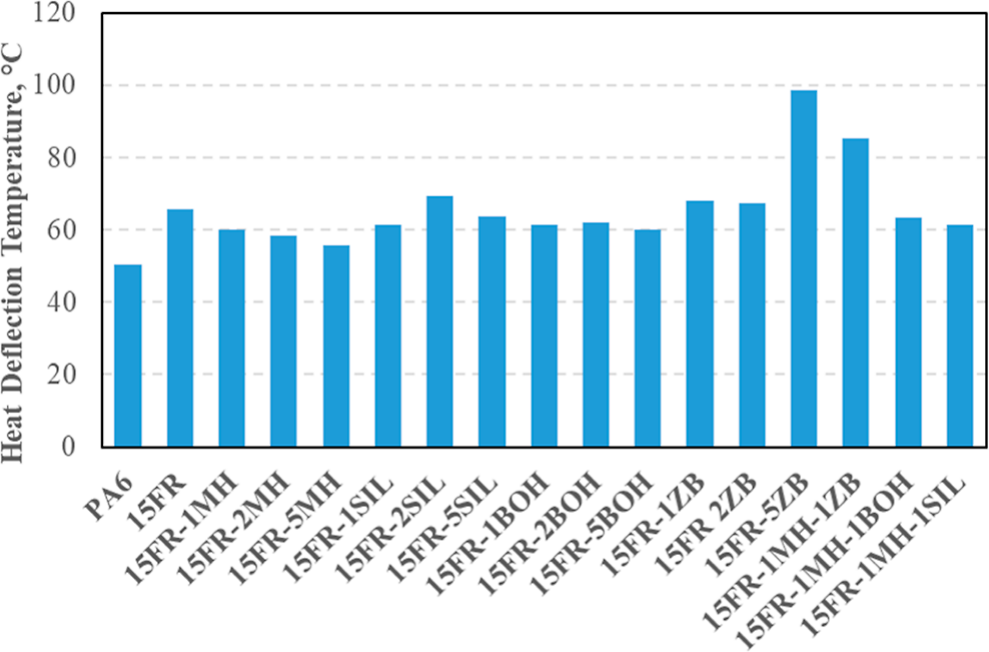
Heat deflection temperatures of PA6-based composites.

Heat deflection temperatures of PA6-based composites are shown in Figure 8. Heat deflection temperature is the measure of the resistance of polymers to deterioration under a certain temperature and load. It is seen from Figure 8 that the addition of mineral synergists to 15 wt % FR-loaded PA6 composites did not cause a change in HDT except for 15 FR-5ZB. The HDT of composites depends on the crystal structure of the matrix, the content of the filler material, and its distribution in the matrix. The increase in HDT temperature, which was observed when only 5 wt % ZB or 1 wt % MH-ZB was added into PA6, could be explained by the rigid structure of the minerals and their homogeneous distribution in the matrix.^34^

### 3.7 Flame Retardant Properties

#### 3.7.1 UL94

Table 6 shows the UL94 flame rating of composites in 1.5 and 3 mm thickness. Neat PA6 has a V2 rating. When 15 wt % of organic phosphonate (OP)-based flame retardant was added to the neat PA6, the flame rating improved to V0. In order to see the synergistic effect of MH, 1 and 2 wt % MH synergists together with 15FR composites have been produced. 15FR-1MH and 15FR-2MH have been shown to be V0, but when 5 wt % MH was used, the V2 rate was seen. With the addition of 1 and 2 wt %, MH could act in a physical way which indicates endothermic dehydration. Therefore, the temperature of the material is decreased owing to the release of water. This leads to the diluted flame by water vapors and a delayed ignition, and the composite reaches the V0 rating.^35,36^

**Table 6:** Flame Retardant Properties of the Samples

samples	UL94 test rating (3 mm thickness)	UL94 test rating (1.5 mm thickness)
PA6	V2	V2
15FR	V0	V0
15FR-1MH	V0	V0
15FR-2MH	V0	V0
15FR-5MH	V2	V2
15FR-1SIL	V0	V0
15FR-2SIL	V0	V0
15FR-5SIL	V0	V2
15FR-1BOH	V0	V0
15FR-2BOH	V0	V0
15FR-5BOH	V0	V0
15FR-1ZB	V0	V0
15FR-2ZB	V0	V0
15FR-5ZB	V0	V0
15 FR-1ZNB-1MH	V0	V0
15FR-1MH-1BOH	V0	V0
15FR-1MH-1SIL	V2	V2

The formation of magnesium oxide (MgO) may act as a protective structure by isolating oxygen from the polymer.^37^ However, a further increase in the MH fraction to 5 wt % leads to more degradation of composites compared to 15FR-1MH and 15FR-2MH, which can also be seen from thermogravimetric analysis (TGA) results. This may reduce viscosity and increase dripping during the UL94 test. It could be concluded that 5 wt % is not proper to ensure the flame retardant properties. The literature was examined considering these results, and it was found that the incorporation of MH at low weight fractions has a positive effect on the UL94 rating, decreasing the total combustion time.^38^ Similar results were seen when 5 wt % SIL was used in the PA6 composites. Although there is a V0 rating for 1.5 mm thickness in 1 and 2 wt % SIL, when the ratio is increased to 5 wt %, it has been observed that the combustion rating of the 1.5 mm thick composite is decreased to V2. The addition of BOH and ZB to the 15FR composite ensures reaching a V0 rating at all weight fractions tested. It is understood that BOH and ZB can exhibit their flame retardant performance together with the flame retardant additive in the material and positively affect the non-flammability properties of the composite. Among hybrid compositions, 15 FR-1ZB-1MH and 15FR-1MH-1BOH have a V0 rating for 3 and 1.5 mm thickness, but 15FR-1MH-1SIL presents a V2 rating, which indicates an improper combination to ensure a V0 rating. The synergistic effect of MH and ZB can be explained by the cooling effect of MH because of endothermic dehydration and increased char formation with ZB. Similarly, it can be interpreted that the flame retardant properties of BOH and MH shorten the burning time of the composites by accelerating char formation. MH and SIL combinations could lead to degradation in composites, and this reduces the viscosity of composites, and a V2 rating could be obtained during the UL94 test.

#### 3.7.2 Glow Wire Flammability Index

GWFI values of the samples at two different thicknesses (2 and 4 mm) are given in Table 7. As it is seen, the GWFI value was found to be 960 °C for all samples except for neat PA6. In the study performed,^38^ it was found that the incorporation of MH, whether coated or not, allows reaching a GWFI of 960 °C, which can be attributed to lower amounts of PA6 in the blend, as well as the formation of a protective structure that insulates the material from the glow wire.

**Table 7:** Glow Wire Flammability Index Values of the Samples

samples	GWFI (2 mm) °C	GWFI (4 mm) °C
PA6	750	750
15FR	960	960
15FR-1MH	960	960
15FR-2MH	960	960
15FR-5MH	960	960
15FR-1SIL	960	960
15FR-2SIL	960	960
15FR-5SIL	960	960
15FR-1BOH	960	960
15FR-2BOH	960	960
15FR-5BOH	960	960
15FR-1ZB	960	960
15FR-2ZB	960	960
15FR-5ZB	960	960
15 FR-1ZNB-1 MH	960	960
15FR-1MH-1BOH	960	960
15FR-1MH-1SIL	960	960

#### 3.7.3 Limiting Oxygen Index

**Figure 9:**
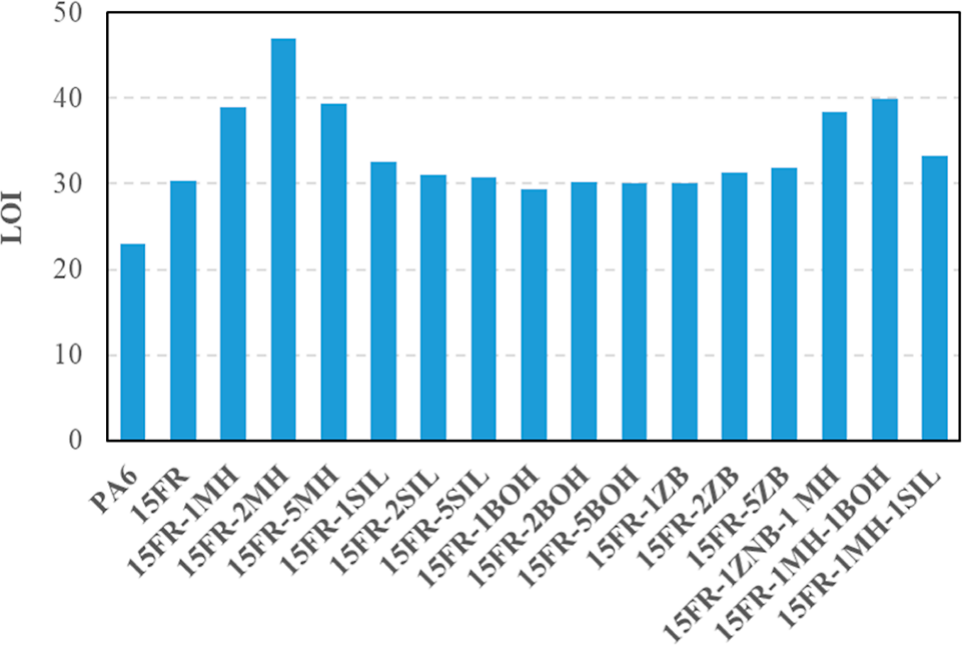
LOI values of the composites.

LOI values of PA6 and its composites are shown in Figure 9. The LOI value of PA6, which is 23, increased to 30 in the case of the 15FR sample without any synergist. When 1 and 2 wt % MH are used, the LOI value increases to 38.9 and 46.9, respectively. However, it was observed that LOI went down to 39.4 in the case of a higher fraction of MH addition. It was understood that considering UL94 ratings, it is proper to use 1 and 2 wt % MH for better flame retardant properties. The SIL synergist has not shown a notable effect on LOI values, presenting a slight increase. When 1 wt % BOH was used, the LOI value decreased from 30.3 to 29.3, and higher fractions of BOH gave smaller LOI values. It could be concluded that BOH is not a proper synergist to increase the LOI value of composites. In the literature, there was a study^14^ made with nano-boehmite and PET polymer by using an in situ method of production, and it was found that boehmite increased the LOI of PET from 18 to greater than 25. The difference in the results observed may be due to the size of the boehmite and the manufacturing technique of the composites. When 1 and 2 wt % ZB were used in the composites, the LOI value was found to be nearly the same as that with the composite without any synergist. However, when ZB loading was increased to 5 wt %, a 6% increase in LOI value was observed. It could be explained that ZB at high fractions could work as a smoke suppressant in flame retarding polymers.^39^ In the study made by,^39^ it was found that the combination of zinc borate with some intumescent flame retardant systems could enhance char formation and improve the char quality, resulting in an improvement in flame retardancy. Another study^40^ found that the LOI values first increase rapidly with the increasing amount of ZB in the PP/IFR composites, but decrease slightly with more than 2 wt % ZB loading. In our study, the obtained results for ZB are similar to those in the literature.^39^ It was concluded that MH is the best synergist to get a high LOI value when it is used alone in the formulation. When hybrid composites are evaluated, 15FR-1ZNB-1 MH, 15FR-1MH-1BOH, and 15FR-1MH-1SIL composites have LOI values of 38.4, 39.9, and 33.3, respectively. Among the formulations that synergist agents were used in together, the incorporation of MH and BOH into the PA6/FR system based on OP exhibited an evident synergistic effect in flame retardant properties and combustion performance of the composites.

#### 3.7.4 Cone Calorimetry Results

The results obtained from the cone calorimeter are presented in Table 8, including PHRR, TTI, THR, tPHRR, TSR, EHC, FIGRA, and MAHRE.

**Table 8:** Results of a Calorimeter Test

samples	PHRR (kW/M^2^)	TTI (S)	THR (MJ/M^2^)	smoke density	TSR (M^2^/M^2^)	EHC (MJ/KG)	FIGRA	MAHRE (kW/M^2^)
PA6	1287	21	154	752	475	28.6	2.84	385
15FR	422	47	127	396	1950	24.6	0.82	241
15FR-1MH	386	43	122	225	1936	25.1	0.78	234
15FR-2MH	314	40	121	141	2003	25.3	0.72	225
15FR-5MH	298	37	117	87	2067	25.4	0.69	216
15FR-1SIL	397	46	123	206	1996	25.2	0.79	236
15FR-2SIL	347	44	123	135	2090	25.2	0.75	226
15FR-5SIL	315	41	125	78	2167	25.4	0.72	221
15FR-1BOH	389	44	126	196	2243	25.3	0.76	222
15FR-2BOH	332	42	127	129	2354	25.3	0.69	205
15FR-5BOH	309	39	126	67	2402	25.2	0.65	197
15FR-1ZB	392	46	129	203	2164	24.9	0.78	225
15FR-2ZB	341	44	128	134	2276	25.1	0.74	219
15FR-5ZB	324	42	128	73	2304	25.2	0.71	204
15 FR-1ZB-1 MH	327	42	127	139	2241	25.1	0.72	221
15FR-1MH-1BOH	321	43	129	135	2301	25.2	0.71	219
15FR-1MH-1SIL	332	45	127	141	2195	25.1	0.74	222

The flammability of the composite containing flame retardants and synergistic fillers is characterized by the TTI in a cone calorimeter. As given in Table 8, the TTI of composites is lower than that of PA6. For PA6 containing flame retardants and/or synergistic fillers, the decrease in comparison to the pure polymer may be explained by an increase in heat absorption on the surface of the sample.^41−43^ For the composite loaded with the mixture of synergistic fillers and FR, a reduction was also observed, possibly due to a mixture of minerals on the surface of samples and the specific effect of flame retardants, facilitating thermal degradation of PA6.

In the case of FR, a reduction of about 67% for PHHR of 15FR compared to the PA6 was obtained. The incorporation of synergistic fillers leads to a decrease in PHRR as a function of synergistic filler loading. The most important and useful parameter in the control of fire hazards, which can be measured by the oxygen depletion technique, is the rate of heat release. It has been known that PHRR depends on the fire scenario and also on the intrinsic properties of plastics.^44,45^

In the case of 15FR, a decrease in EHC of the composites was observed, indicating that FR acts both in the gas and condensed phase. Braun et al. (2007) have noted that aluminum phosphonate in polyamide acts as a flame inhibitor and releases phosphoric acid. This released phosphoric acid can promote the formation of char.^9^

As shown in Table 8, the THR of the 15FR composites decreased from 154 MJ/m^2^ (PA6) to 127 MJ/m^2^, respectively. The THR is the integral of the HRR and corresponds to the radiant flux levels.^46^ At the same radiant flux, the THR depends on several factors, such as the effective heat of combustion of the volatiles, the combustion efficiency, and the total mass loss in the flame zone.^45^

As given in Table 8, both FIGRA and MAHRE of the 15FR and other composites decreased. The decreases in FIGRA and MAHRE indicate that the composites have a better fire performance compared to that of pure PA6. The FIGRA of 15FR decreased from 2.91 to 0.82 compared to that of virgin PA6. One can say that PA6 composites with FR/synergistic materials have better fire performance compared to PA6.^47^ Smoke density values of PA6, 15FR, 15FR-1MH, 15FR-2MH, 15FR-5MH, 15FR-1SIL, 15FR-2SIL, 15FR-5SIL, 15FR-1BOH, 15FR-2BOH, 15FR-5BOH, 15FR-1ZB, 15FR-2ZB, 15FR-5ZB, 15FR-1MH-1ZB, 15FR-1MH-1BOH, and 15FR-1MH-1SIL were obtained to be 752, 396, 225, 141, 87, 206, 135, 78, 196, 129, 67, 203, 134, 73, 139, 135, and 141. It is seen that MH, ZB, BOH, and SIL decrease the smoke density values of PA6-based composites. The concentrations of CO and CO_2_ gases and CIT values of samples are presented in Table 9. It is seen that CIT values of all FR-loaded PA6 composites are lower than 1.5. PA6-15FR-5ZB shows the lowest CIT value at 480 s. The concentrations of CO and CO_2_ gases in PA6 decreased after FR loading. ZB, MH, and BOH loading has led to more decrease in the concentrations of CO and CO2. PA6-15FR-5ZB and PA6-15FR-5MH exhibited the lowest CO and CO_2_ concentrations, respectively. It is known that the steam produced from the thermal decomposition of MH could dilute flammable gases, form a layer of adiabatic material on the interface of the plastic material and flame, and have the effect of preventing the fire from spreading.^12,13^ Similarly, thermal decomposition of boehmite (AlOOH) into water vapor and aluminum oxide takes place, and water vapor dilutes the flammable gas concentration and limits the accessibility of oxygen to the surface of the composite.^48^ Moreover, it is expected that the oxide layer acts as a barrier, protecting the polymer against further decomposition. Upon heating and polymer combustion, ZB is subjected to endothermic dehydration, and elimination of the chemically bonded water molecules occurs; thus, water vapors provide a heat sink, delay the combustion, and dilute the concentration of the oxygen and gaseous flammable components, leading to an increase in the residual char formation.^49^ In addition to those, high molecular-weight SIL, which is known for its flame retardant synergist and smoke suppressant properties, led to lower smoke density values. It can also be noted that when these additives are used in a hybrid form, different mechanisms are expected to work effectively together during the combustion process.

**Table 9:** Concentrations of Gases and CIT Values of the Samples

sample	CIT	CO (%)	CO_2_ (%)
PA6	1.75	0.71	1.81
PA6-15FR	0.66	0.42	1.38
PA6-15FR-1MH	0.59	0.36	1.20
PA6-15FR-2MH	0.56	0.33	1.18
PA6-15FR-5MH	0.48	0.27	0.97
PA6-15FR-1SIL	0.69	0.47	1.44
PA6-15FR-2SIL	0.62	0.39	1.38
PA6-15FR-5SIL	0.60	0.38	1.34
PA6-15FR-1BOH	0.58	0.35	1.32
PA6-15FR-2BOH	0.57	0.34	1.27
PA6-15FR-5BOH	0.51	0.28	1.22
PA6-15FR-1ZB	0.50	0.27	1.20
PA6-15FR-2ZB	0.49	0.28	1.20
PA6-15FR-5ZB	0.43	0.23	0.99
PA6-15FR-1MH-SIL	0.63	0.40	1.40
PA6-15FR-1MH-1BOH	0.58	0.34	1.29
PA6-15FR-1MH-1ZB	0.52	0.29	1.03

### 3.8 SEM Morphological Observation

**Figure 10:**
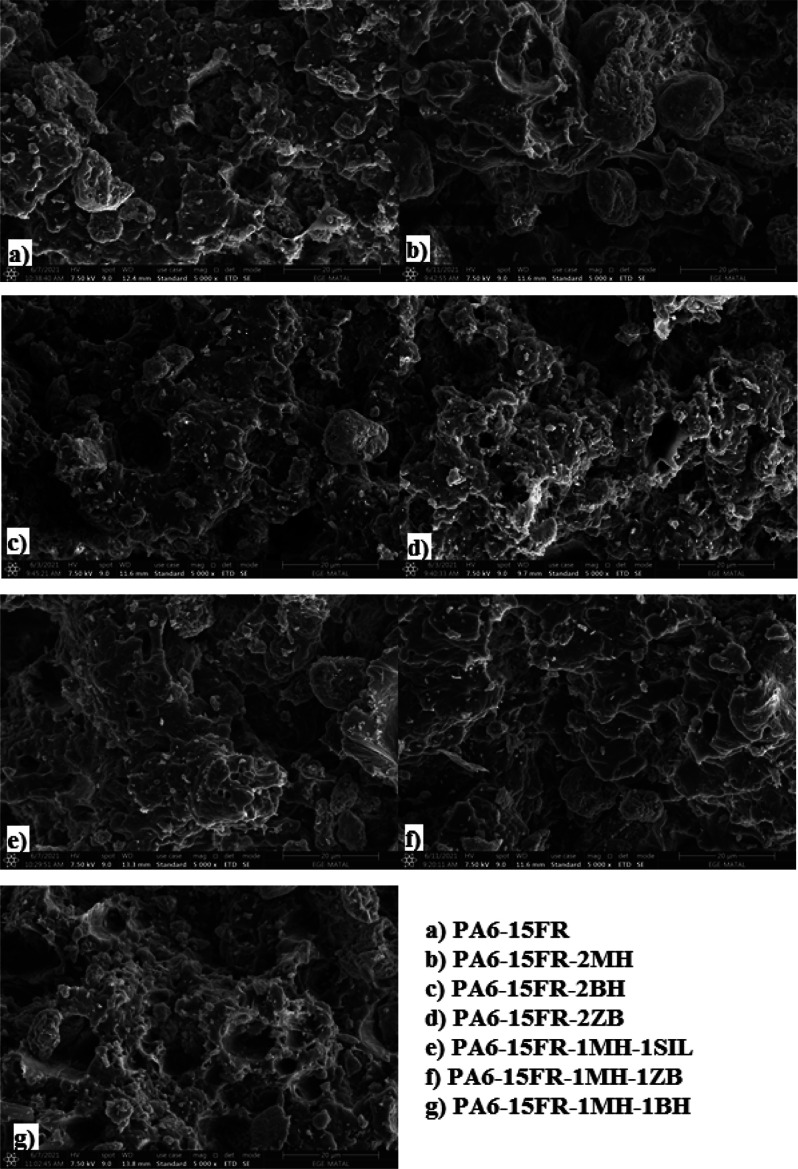
SEM micrographs of PA6 and its composites.

Figure 10a–g show SEM micrographs of fractured sections of composites. As can be seen from Figure 10, FR particles were distributed homogeneously within the matrix. Figure 10a–h show that the dispersed particles are about 1 μm, and various particle sizes are distributed within the matrix. The defects resulting from the particle pulling out of the matrix at different sizes can be seen in Figure 10c–g, which indicates poor adhesion between particles and the polymer matrix.^50^

## 4 Conclusions

In this work, mechanical, thermal, physical, and flame retardant properties of PA6 composites filled with halogen-free flame retardants and synergistic materials were studied. Especially, the fire performance of the composites was investigated by LOI, glow wire flammability index, UL94, and cone calorimetry tests. The incorporation of MH (alone) or MH-BOH (hybrid) into PA6/FR resulted in the highest LOI value in composites, indicating a better combustion performance among all formulations. The obtained GWFI value of 960 °C for all composites except for virgin PA6 proved the formation of a protective layer that insulates the sample from the glow wire. In the case of using synergistic materials, PA6-based composites presented a flame rating of V0 for all combinations except 5MH (alone) and 1MH-1SIL (hybrid). The ignitability, THR rate, fire growth rate, and TSR results showed that the halogen-free flame retardants together with metal synergist additives have great potential to enhance the fire performance of PA6-based composites. It was determined that 15FR-2MH, 15FR-5MH, 15FR-2SIL, 15FR-15SIL, 15FR-2BOH, 15FR-5BOH, 15FR-22ZB, 15FR-5ZB, 15FR-1MH-1ZB, 15FR-1MH-1BOH, and 15FR-1MH-1SIL showed required smoke density values for R22 according to the EN45545 standard. However, 15FR-2MH, 15FR-5MH, 15FR-1MH-1ZB, 15FR-1MH-1BOH, and 15FR-1MH-1SIL composites exhibited both the required smoke density and LOI values for R22. It can be said that hybrid synergists provide all requirements according to R22.
